# A Synthesized Heuristic Task Scheduling Algorithm

**DOI:** 10.1155/2014/465702

**Published:** 2014-09-01

**Authors:** Yanyan Dai, Xiangli Zhang

**Affiliations:** Institute of Information and Communication, Guilin University of Electronic Technology, Guilin 541004, China

## Abstract

Aiming at the static task scheduling problems in heterogeneous environment, a heuristic task scheduling algorithm named HCPPEFT is proposed. In task prioritizing phase, there are three levels of priority in the algorithm to choose task. First, the critical tasks have the highest priority, secondly the tasks with longer path to exit task will be selected, and then algorithm will choose tasks with less predecessors to schedule. In resource selection phase, the algorithm is selected task duplication to reduce the interresource communication cost, besides forecasting the impact of an assignment for all children of the current task permits better decisions to be made in selecting resources. The algorithm proposed is compared with STDH, PEFT, and HEFT algorithms through randomly generated graphs and sets of task graphs. The experimental results show that the new algorithm can achieve better scheduling performance.

## 1. Introduction

A heterogeneous computing system (HCS) is a computing platform with diverse sets of interconnected resources via high speed network to execute parallel and distributed applications. Due to diverse computational resources, the efficiency of an application on the available resources is one of the key factors for achieving high performance computing. The general form of task scheduling problem in HCS can be represented as directed acyclic graph (DAG). The common objective of scheduling is to assign tasks onto suitable resources and order their execution so that task precedence requirements are satisfied with a minimum schedule length [1]. The scheduling problem is shown to be NP-complete [2–4], so it is expected to be solved by heuristic algorithm.

A typical task scheduling model is based on a directed acyclic graph, including static and dynamic scheduling. Heuristic algorithm is static scheduling algorithm, which is composed of duplication-based algorithms, clustering algorithms, and list scheduling algorithms [3]. List scheduling algorithms are widely used for their high scheduling efficiency and simple design idea. The basic idea of list scheduling is to construct a schedule list by assigning priority for each task, and tasks are selected to a processor which minimizes the execution time. Classical examples of list scheduling algorithms were proposed in [5–10]. The heterogeneous earliest finish time (HEFT) [5] uses a recursive procedure to compute the rank of a task by traversing the graph upwards from the exit task and vice-versa for critical path on a processor (CPOP) [5]. Improvement heterogeneous earliest finish time (IHEFT) [6] acquires a better task list by changing the task's upward weight calculation method. Stand deviation-based algorithm for task scheduling (SDBATS) [7] uses the standard deviation of the expected execution time of a given task on the available resources as a key attribute for assigning task priority. In [8], the proposed algorithm determines the critical path of the task graph and selects the next task to be scheduled in a dynamic fashion. Predict earliest finish time (PEFT) [9] algorithm is only based on computation of an optimistic cost table (OCT) that is used to rank tasks and for resource selection. A novel algorithm named the longest dynamic critical path (LDCP) [10] assigns priorities by considering the critical path attribute of the given DAG.

The idea of task duplication is to try to duplicate the parents of the current selected task onto the selected resource or on to the other resources, aiming to reduce or optimize the task finish time. Many duplication-based algorithms are proposed in recent years; for example, in [11], a path priority-based heuristic task scheduling algorithm was studied. Heterogeneous critical task (HCT) [12] scheduling algorithm defines the critical task and the idle time slot. Selected task duplication for heterogeneous (STDH) [13] duplicates the parent tasks for advancing the earliest starting time of the current candidate task to reduce the inter processor communication cost. Heterogeneous earliest finish time with duplication (HEFD) [14] uses task variance as computation capacity heterogeneity factor for setting weights to tasks and edges. The algorithm in [15] is a three-phase algorithm with a dynamic phase to assign a priority to each task. To search and delete redundant task duplications dynamically in the process of scheduling, a novel resource-aware scheduling algorithm with duplications (RADS) was proposed in [16]. There are some researches such as [17, 18] combining DVS technique with duplication strategy to reduce energy consumption. The algorithms proposed in [19, 20] are designed to solve the problem of resource waste. Other duplication-based algorithms such as selective duplication (SD) [21] and heterogeneous critical parents with fast duplicator (HCPFD) [22] are also worth studied.

Clustering algorithms merge tasks in a DAG to an unlimited number of clusters, and tasks in a cluster are allocated on the same resource. Some classical examples are cluster mapping algorithm (CMA) [23], clustering and scheduling system (CASS) [24], objective flexible clustering algorithm (OFCA) [25], and so on. In [26, 27], two novel algorithms were proposed, but they have limitations in higher heterogeneity systems. To reduce energy consumption without increasing the schedule length, [28] reclaims both static and dynamic slack time and employs different frequency adjusting techniques in different slack time, and [29] studies the slack time for noncritical jobs, extends their execution time, and reduces the energy consumption without increasing the task's execution time as a whole. Additionally, these methods are based on DAG scheduling: heterogeneous critical path first synthesized (HCPFS) [30], heterogeneous select value (HSV) [31], heterogeneous chip multiprocessor global comparatively optimum task scheduling (HGCOTS) [32], and so on.

The HEFT [5] algorithm has two phases: the task prioritizing phase and the processor selection phase. In the first phase, a task list is generated by sorting the tasks in decreasing order of the upward rank. In the processor selection phase, tasks are scheduled to the best processor that minimizes the task's finish time. However, the algorithm does not take into account the critical task and interprocessor communication cost impact on the whole task graph scheduling time. Task scheduling efficiency is not high which still should be optimized further.

The PEFT [9] algorithm is based on the computation of an optimistic cost table (OCT) on which task priority and processor selection are based. The OCT is a matrix in which the rows indicate the number of resources, where each element OCT(*t*_*i*_, *r*_*j*_) indicates the maximum of the shortest paths of *t*_*i*_ children's tasks to the exit node considering that resource *r*_*j*_ is selected for task *t*_*i*_. The algorithm has the same time complexity as the HEFT and SDBATS algorithms, that is, *O*(*n* ^2^ · *m*) for *n* tasks and *m* resources. But the algorithm does not take into account the critical tasks, which decide the whole DAG scheduling time. So this algorithm delays the scheduling length to some extent.

The STDH [13] algorithm duplicates the parent tasks for advancing the earliest starting time of the current candidate task to reduce the interprocessor communication cost. By this way, the overall run time of application is shortened, but the algorithm scheduled only by one attribute. In the case of that different tasks should have the same priority, it cannot work well. Therefore, in order to evaluate the priority of tasks reasonably, multiple attributes are more suitable for sorting tasks.

In this paper, we propose a synthesized heuristic task scheduling algorithm based on both duplication-based techniques and list-based approach for heterogeneous computing systems. The HCPPEFT algorithm has two phases, the task prioritizing phase and the resource selection phase (see Algorithm 1). In the first phase, we suggest a new approach of critical task, the tasks with longer path to exit task, and the number of predecessors to construct the scheduling queue. In the second phase, the duplication of tasks is optimized for all immediate parent tasks and look ahead policies is to guarantee the tasks ahead will finish earlier. The rest of the paper is organized as follows. In Section 2, we define the task scheduling problem and discuss some basic attributes of DAG scheduling.  Section 3 introduces HCPPEFT algorithm and in Section 4 the results of the experiment are discussed.  Section 5 concludes the paper.

## 2. Problem Definition

### 2.1. Task Model

A scheduling system model is composed of an application in heterogeneous computing environment. An application is represented by *G* = (*T*, *E*), where *T* is the set of *t* tasks and *E* is the set of e edges between the tasks. Each edge *e*_*ij*_ ∈ *E* represents the precedence constraint such that task *t*_*i*_ should complete its execution before task *t*_*j*_ starts. Communication cost required to be transmitted from task *t*_*i*_ to task *t*_*j*_ is represented by a *C*(*t* × *t*) matrix, in which each *c*_*ij*_ gives the amount of data on *e*_*ij*_ edge. The heterogeneous system consists of a set of resources *R* = {*r*_1_, *r*_2_,…, *r*_*m*_} of *m* independent resources fully connected by a high speed network. A computation cost matrix is represented as *W*(*t* × *m*), in which each *w*_*i*,*j*_  gives the estimated time to execute *t*_*i*_  on resource *r*_*j*_. We further assume that any two connecting tasks scheduled on the same resource have zero communication costs.

### 2.2. DAG Scheduling Attributes

Before proceeding to the next section, it is necessary to discuss some scheduling attributes such as rank upward, rank downward, earliest start time, earliest finish time, and optimistic cost table, which will be used in the proposed scheduling algorithm.

**Definition 1 .**

A task with no predecessors is called an entry task (*t*_entry_), and pred(*t*_*i*_) denotes the set of immediate predecessors of task *t*_*i*_ in a given task graph. Similarly, a task with no successors is called an exit task (*t*_exit_), and succ(*t*_*i*_) denotes the set of immediate successors of task *t*_*i*_ in a given task graph. If a task graph has multiple entry or exit nodes, a dummy entry or exit node with zero communication edges and zero weight can be added to the graph.

**Definition 2 .**

The upward rank of a task *t*_*i*_ is calculated using the following equation:

(1) $$\begin{array}{c} {\mathrm{rank}}_{u}\left(t_{i}\right)=\bar{w_{i}}+\underset{t_{j}\in \text{succ}\left(t_{i}\right)}{\max}\left\{\bar{c_{ij}}+{\mathrm{rank}}_{u}\left(t_{j}\right)\right\}, \end{array}$$

where $\bar{w_{i}}$ is the average computation cost of task *t*_*i*_, and $\bar{c_{ij}}$ is the average communication cost of the edge from task *t*_*i*_ to task *t*_*j*_. For the exit task *t*_exit_, upward rank value is

(2) $$\begin{array}{c} {\mathrm{rank}}_{u}\left(t_{\text{exit}}\right)=\bar{w_{\text{exit}}}. \end{array}$$

Similarly, the downward rank of task *t*_*i*_ is defined by the following equation:

(3) $$\begin{array}{c} {\mathrm{rank}}_{d}\left(t_{i}\right)=\underset{t_{j\in \text{pred}(t_{i})}}{\max}\left(\bar{w_{j}}+\bar{c_{ji}}+{\mathrm{rank}}_{d}\left(t_{j}\right)\right). \end{array}$$

For the entry task *t*_entry_, the downward rank value is equal to zero.

**Definition 3 .**

EST(*t*_*i*_, *r*_*j*_) and EFT(*t*_*i*_, *r*_*j*_) are the earliest start time and earliest finish time of a task *t*_*i*_ on the resource *r*_*j*_ and are defined by

(4) EST(ti,rj)  =max⁡{avail(ti,rj),max⁡tm∈pred(ti){AFT(tm)+cmi}},

(5) $$\begin{array}{c} \text{EFT}\left(t_{i},r_{j}\right)=w_{i,j}+\text{EST}\left(t_{i},r_{j}\right), \end{array}$$

where avail(*t*_*i*_, *r*_*j*_) is the earliest time at which resource *r*_*j*_ is ready for task execution, AFT(*t*_*m*_) is actual finish time of task *t*_*i*_, and *c*_*mi*_ is communication cost of the edge from task *t*_*m*_ to task *t*_*i*_. Before computing earliest finish time of a task *t*_*i*_, all the immediate predecessor tasks of *t*_*i*_ must have been scheduled. For the entry task *t*_entry_, EST(*t*_entry_, *r*_*j*_) = 0.

**Definition 4 .**

If the task start execution time depends on the arrival time of parent tasks, there will be a free time slot, that is, the task to wait for the time of arrival of the parent task, can be defined as

(6) $$\begin{array}{c} \text{slot}\left(t_{i},r_{j}\right)=\text{EST}\left(t_{i},r_{j}\right)-\text{avail}\left(t_{i},r_{j}\right). \end{array}$$

**Definition 5 .**

The OCT is a matrix in which the rows indicate the number of tasks and the columns indicate the number of resources, where each element OCT(*t*_*i*_, *r*_*j*_) represents the maximum optimistic processing time of the children of task *t*_*i*_. The OCT value of task *t*_*i*_ on resource *r*_*j*_ is recursively defined by

(7) $$\begin{array}{c} \text{OCT}\left(t_{i},r_{j}\right)=\underset{t_{j}\in \text{succ}\left(t_{i}\right)}{\max}\left[\underset{r_{k}\in r}{\min}\left\{\text{OCT}\left(t_{j},r_{k}\right)+w_{j,k}+c_{ij}\right\}\right]. \end{array}$$

**Definition 6 .**

The schedule length (makespan) of the task graph denotes the finish time of the last task and is defined as follows:

(8) $$\begin{array}{c} \text{makespan}=\max\left\{\text{AFT}\left(t_{\text{exit}}\right)\right\}. \end{array}$$

The objective function of the task scheduling problem is to determine an assignment of tasks of a given task graph to resources, such that its schedule length is minimized, which satisfying all precedence constraints.

## 3. The Proposed Algorithm HCPPEFT

### 3.1. Task Prioritizing Phase

**Figure 1:**
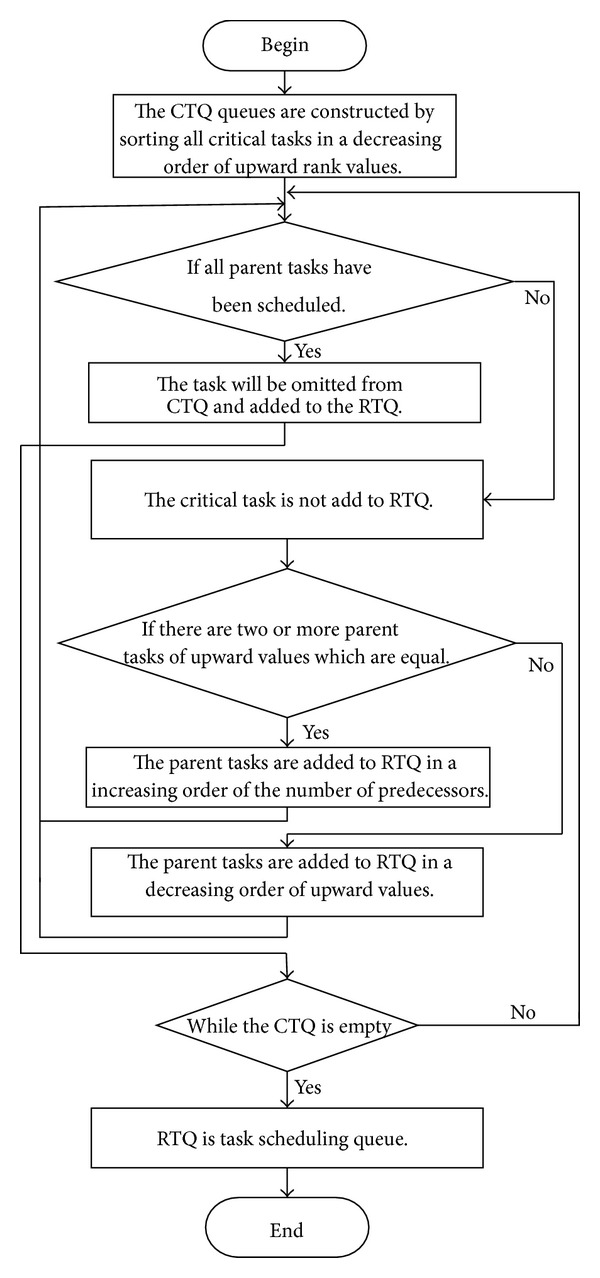
Constructing task scheduling queues.

The critical path is a path with the longest execution time in directed acyclic graph, which of task play a decisive role to finish time. In the process of task scheduling, critical task is assigned the highest priority to make it firstly scheduled. However, critical task usually have one or more parent tasks. If parent tasks cannot get a reasonable scheduling, the start execution time will be delayed. Therefore it not only assigns besides the critical tasks the highest priority, but also needs the parent tasks of critical tasks to allocate higher-priority. Immediate parent tasks are sorted in a decreasing order of rank upward values. If the rank upward values are equal, select the tasks with respect to the predecessors increase. In order to construct the task scheduling queue, two empty queues of tasks are constructed firstly, which are CTQ and RTQ. CTQ is used to store critical task and RTQ is used to store the task scheduling ready queue. The process of constructing task scheduling queues is expressed as Figure 1. The detailed explain is presented as follows.

1. Upward rank (rank⁡_*u*_), downward rank (rank⁡_*d*_), and the summation of upward and downward ranks (rank⁡_*u*_ + rank⁡_*d*_) values for all tasks are computed.
2. The critical path length is equal to the entry task's priority. The entry task is marked as a critical path task. If CT(*t*_*i*_) = rank⁡_*u*_(*t*_*i*_) + rank⁡_*d*_(*t*_*i*_) for each task *t*_*i*_ in DAG, then CT(*t*_*i*_) = CT(*t*_entry_), where *t*_entry_ is the entry task, CT = {*t*_entry_} is the set of tasks on the critical path. The all critical path task will be added to the CTQ by decreasing order of rank⁡_*u*_.
3. The CTQ at the beginning contains all critical tasks. In fact a critical task will be omitted from the CTQ and added to the RTQ after all parent tasks are added to the RTQ. The priority of the parent tasks is determined based on their rank⁡_*u*_. The parent task with the highest rank⁡_*u*_ gets high priority.
4. If there are two or more parent tasks of rank⁡_*u*_ are equal. The priority of the parent tasks is generated by increasing order of the number of predecessors.
5. The above steps are executed until the CTQ queue is null. The RTQ queue is task scheduling queue.

**Figure 2:**
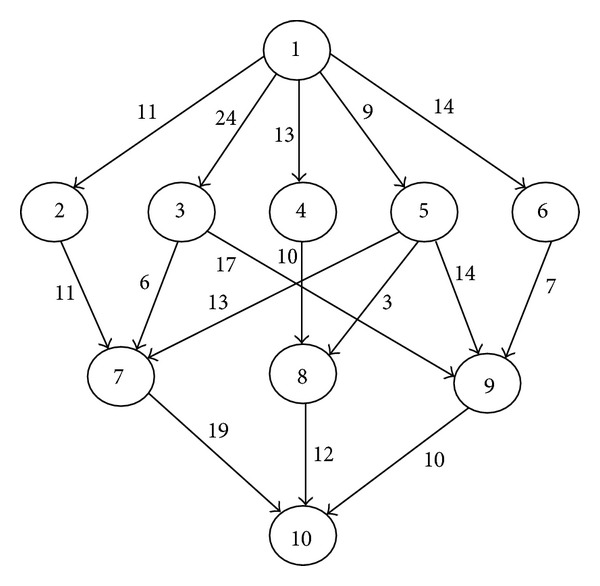
A sample task graph with 10 tasks.

To illustrate how the task prioritizing queue is constructed, a random task graph with 10 tasks and 16 edges is shown in Figure 2. The corresponding computation cost with respect to each resource (i.e., {*r*_1_, *r*_2_^  ^  , *r*_3_}) is presented in Table 1. Based on the values in Table 2, the critical path in Figure 2 is {*t*_1_, *t*_3_, *t*_7_, *t*_10_}. The all critical tasks will be added to the CTQ by decreasing order of rank upward values; that is, CTQ = {*t*_1_, *t*_3_, *t*_7_, *t*_10_}. Because the entry task *t*_1_ has no parent task, it will be omitted from the CTQ and added to the RTQ immediately. After that, task *t*_3_ is added in RTQ and deleted from CTQ. Until parents tasks *t*_2_ and *t*_5_ have been selected, the critical task *t*_7_ can be selected. Till now, we can get CTQ = {*t*_10_} and RTQ = {*t*_1_, *t*_3_, *t*_5_, *t*_2_, *t*_7_}. Before the critical task *t*_10_ is selected, the parent tasks *t*_8_ and *t*_9_ must have been scheduled. But the rank upward values of parent tasks *t*_8_ and *t*_9_ are equal, selecting tasks with respect to the predecessors increase. Task *t*_8_ is selected and then is task *t*_9_. At this time, the scheduling order of remaining tasks is {*t*_4_, *t*_8_, *t*_6_, *t*_9_, *t*_10_}. Finally, the task queue is constructed, which is {*t*_1_, *t*_3_, *t*_5_, *t*_2_, *t*_7_, *t*_4_, *t*_8_, *t*_6_, *t*_9_, *t*_10_}.

### 3.2. Resource Selection Phase

In this phase, tasks are assigned to the resources and duplication is employed to minimize the finish time. To start execution on a resource, the task *t*_*i*_ has to wait for the data arrivals from all of its immediate parents, so as to meet precedence constraints. The parent task *t*_*m*_ of task *t*_*i*_ whose schedule on different resources and whose data arrival time at task *t*_*i*_ is the latest parent task. Duplication condition [13] is defined as

(9) $$\begin{array}{c} \text{slot}\left(t_{i},r_{j}\right)\geq w_{m,j},\\ \text{EFT}\left(t_{m},r_{j}\right)<\text{EST}\left(t_{i},r_{j}\right). \end{array}$$

If it is satisfied, then the parent task *t*_*m*_ will be duplicated on the resource that *t*_*i*_ assigned to. Simultaneously compute the earliest finish time by (5) and update the free time slots by (6). It is to be noted that the best-suited resource may not be achieved if we followed by (5). Because the best scheduling consider not only the current task's gain in complete time but also the gain in a sequence of tasks. With computation of an OCT by (7) not only cannot increase the time complexity but also guarantee that the tasks ahead will finish earlier. In this way, we compute the optimistic EFT (*O*_EFT_), which sums to EFT the computation time of the longest path to the exit task, to select a best-suited resource. *O*_EFT_ is defined as

(10) $$\begin{array}{c} O_{\text{EFT}}\left(t_{i},r_{j}\right)=\text{EFT}\left(t_{i},r_{j}\right)+\text{OCT}\left(t_{i},r_{j}\right). \end{array}$$

For a given DAG, the time complexity of scheduling algorithm is usually expressed in terms of number of tasks *n* and number of resources *m*. The time complexity of STDH is of constructing schedule queue *O*(*n* ^2^). The resource selection for all tasks can be done in time complexity *O*(*n* ^2^ · *m*). The total time is *O*(*n* ^2^ · *m*). PEFT requires the computation of an OCT table is *O*(*m*(*n* ^2^ + *n*)), and to assign the tasks to resources, the time complexity is of the order *O*(*n* ^2^ · *m*). The total time is *O*(*n* ^2^ · *m*). The HEFT algorithm has a *O*(*n* ^2^ · *m*) complexity for *n* tasks and *m* resources. The HCPPEFT algorithm requires the construing task scheduling queue is *O*(*n* ^2^ · *m*), and the time complexity of resource select phase is *O*(*m*(*n* ^2^ + *n*)). That is, the HCPPEFT algorithm has the same time complexity as the STDH, PEFT, and HEFT algorithms.

### 3.3. HCPPEFT Algorithm Implementation

To evaluate the performance of the HCPPEFT algorithm, we implement this algorithm on the PC platform using MATLAB software.

A randomly generated task graph is shown in Figure 2 firstly. Then the corresponding computation costs are presented in Table 1. By calculating (7), the OCT values are shown in Tables 3 and 4 which give an example that demonstrates the HCPPEFT for the DAG of Figure 2 in the end.

**Figure 3:**
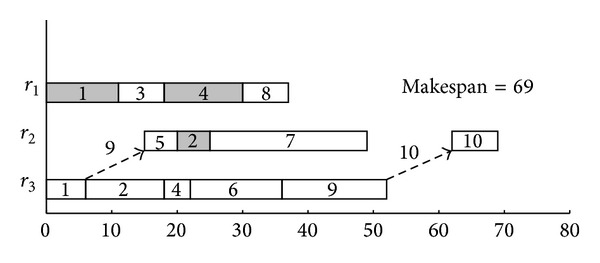
Schedule of task graph with the HCPPEFT algorithm.

As an illustration, Figure 3 presents the schedules obtained by the HCPPEFT algorithm, where the gray blocks with numbers are the duplicated tasks. The schedule length, which is equal to 69, is shorter than that of the related algorithms; specifically, the schedule lengths of STDH, PEFT, and HEFT Algorithm are 73, 78, and 77, respectively.

## 4. Experimental Results and Discussion

This section presents a performance comparison of the proposed algorithm HCPPEFT with four well-known task scheduling algorithms such as STDH, PEFT, and HEFT by randomly generated task graph and sets of task graphs. Three metrics are used for performance evaluation.

### 4.1. Comparisons Metrics 

#### 4.1.1. Scheduling Length Ratio

Since a large set of task graphs with different properties is used, it is necessary to normalize the schedule length to the lower bound, which is called the schedule length ratio (SLR). SLR is defined as follows:

(11) $$\begin{array}{c} \text{SLR}=\frac{\text{makespan}}{\sum_{t_{i}\in {\text{CP}}_{\min}}\min_{r_{j}\in R}\left\{w_{\left\{i,j\right\}}\right\}}, \end{array}$$

where CP_min⁡_ is the minimum computation cost of the critical path tasks in SLR. There is no makespan less than the denominator of the SLR equation since the denominator is the lower bound. Therefore, the algorithm with the lowest SLR is the best algorithm with respect to performance.

#### 4.1.2. Speedup

The speedup value for a given task graph is defined as the ratio of sequential execution times to the parallel execution times. The sequential execution time is computed by assigning all tasks to a single resource that minimizes the computation costs. Speedup is defined as follows:

(12) $$\begin{array}{c} \text{speedup}=\frac{\min_{r_{j}\in R}\left\{\sum_{t_{i}\in T}w_{i,j}\right\}}{\text{makespan}}. \end{array}$$

#### 4.1.3. Efficiency

In the general case, efficiency is defined as the ratio of the speedup value to the number of resources used in schedule task graph. Efficiency is defined as follows:

(13) $$\begin{array}{c} \text{Efficiency}=\frac{\text{speedup}}{\text{number}\;\;\text{of}\;\;\text{resource}}. \end{array}$$

### 4.2. Randomly Generated Task Graph and Performance Comparison

To evaluate the performance of the HCPPEFT algorithm, we first considered randomly generated task graphs. For this purpose, a random graph generator available at [13] was implemented to generate a variety of weighted graphs with various characteristics. The input parameters of the generator are the communication to computation ratio (CCR), number of tasks, out degree of a node (out_degree), edge weight, node weight, and number of resources. Our simulation framework first generate a large set of random task graphs with different characteristics, which is followed by the execution of the task scheduling algorithms, and finally, it computes the performance metrics.

The performance of the HCPPEFT, STDH, PEFT, and HEFT algorithms is compared with respect to various graph characteristics according to SLR, speedup, and efficiency.

**Figure 4:**
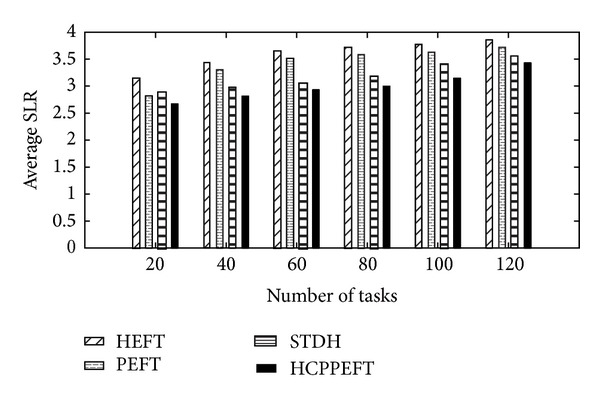
Average SLR with CCR = 2 for random task graphs.

**Figure 5:**
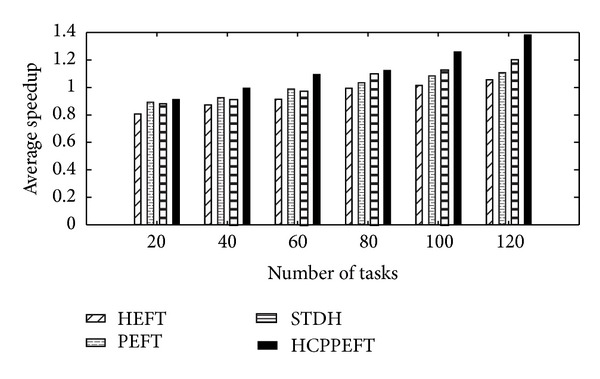
Average speedup with CCR = 2 for random task graphs.

Firstly, we have evaluated the performance of the algorithm with respect to various numbers of tasks and the number of resources was considered as a fixed value of 10. Each value of the experimental results is the average of the results produced from 200 different random task graphs. In these experiments CCR and out_degree were considered as fixed value of 2 and 5, and *n* was restricted to the following values: *n* ∈ {20, 40, 60, 80, 100, 120, 150, 200}. The edge weight is generated randomly from 1 to 300, as the node weight is 1 to 30. In Figures 4 and 5, the simulation results show that the new algorithm outperforms the other algorithms according to the SLR and speedup. The average SLR value of the HCPPEFT algorithm is better than the HEFT algorithm by 19.99%, the PEFT algorithm by 14.43%, and the STDH algorithm by 5.72%; and the average speedup value of the HCPPEFT algorithm is better than the HEFT algorithm by 16.33%, the PEFT algorithm by 10.79%, and the STDH algorithm by 8.69%.

**Figure 6:**
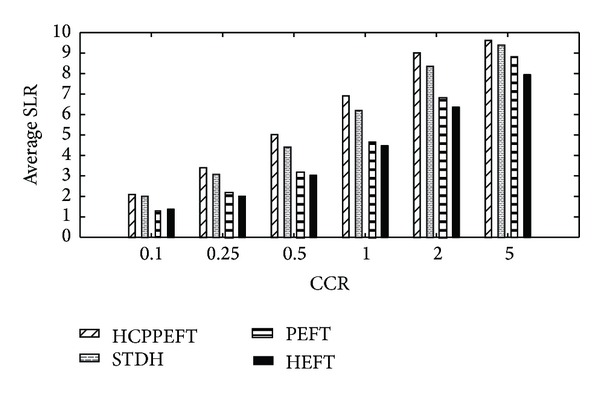
Average SLR with 100 nodes for random task graphs.

**Figure 7:**
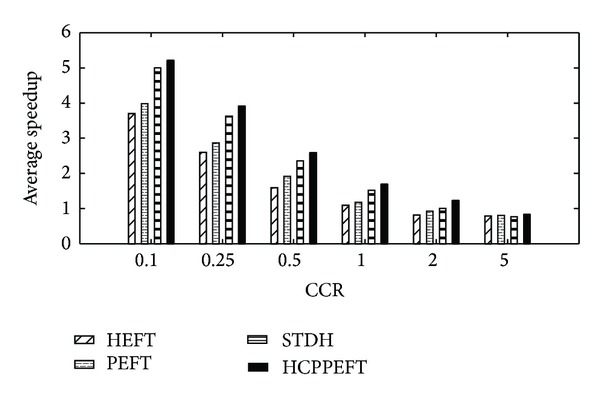
Average speedup with 100 nodes for random task graphs.

The next experiment is with respect to CCR increment. Each value of the experimental results is the average of the results produced from 200 different random task graphs. In each graph, the CCR is randomly selected from 0.1, 0.25, 0.5, 1, 2, and 5, and the node weight is generated randomly from 1 to 30, as the edge weight is 1 to 300. Also *m*, *n*, and out_degree are fixed to 10, 100, and 5, respectively. The results are shown in Figures 6 and 7. In comparison to the HEFT, PEFT, and STDH algorithms on all the generated graphs, the average SLR value obtained by the HCPPEFT algorithm is better by as much as 43.35%, 32.83%, and 7.08%, respectively; and the average speedup value is better by as much as 31.62%, 24.56%, and 7.72%, respectively. This improvement is due to the duplication phase and predicting earliest finish time. The communication cost can reach its lowest ratio by duplication with respect to the increment of CCR; therefore the new algorithm will have great improvement. On the other hand, we can select the best-suited resource that achieves a shorter finish time for the tasks in the next steps by forecasting the impact of an assignment for all children of the current task.

**Figure 8:**
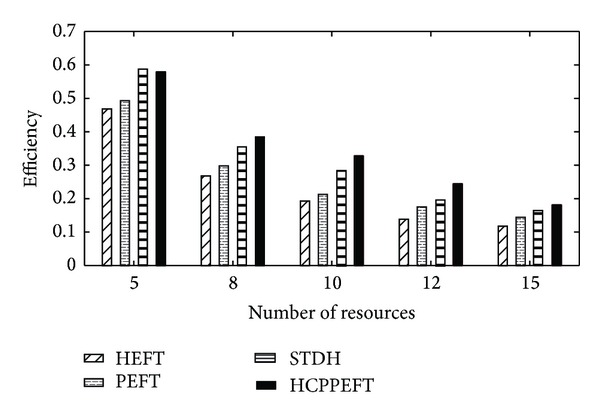
Efficiency for different numbers of resources.

The last set of experiments compare the performance of the algorithm as the value of resource numbers increase. Each value of the experimental results is the average of the results produced from 200 different random task graphs. In these experiments CCR, *n*, and out_degree were fixed to 0.5, 150, and 5, respectively, and the edge weight is generated randomly from 1 to 300 and the node weight is 1 to 30. The number of resources was restricted to the following values: *m* ∈ {5, 8, 10, 12, 15}. In Figure 8, the simulation results show that the HCPPEFT algorithm outperforms the HEFT, PEFT, and STDH algorithms by 30.98%, 22.95%, and 7.45%, respectively. As was expected, the average scheduling length of HCPPEFT, HEFT, PEFT, and STDH algorithms is reduced as the number of resources increases. This decrement is due to parallelism characteristics.

### 4.3. Sets of Task Graphs and Performance Results

**Figure 9:**
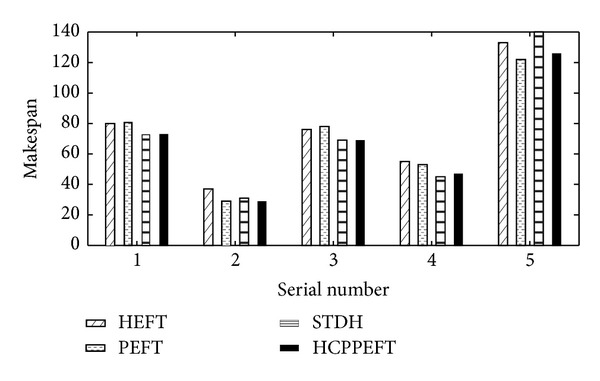
Makespan with HEFT, PEFT, STDH, and HCPPEFT for the sets of task graphs.

In addition to randomly generated task graph, we also use the sets of task graphs to evaluate the performance of the new algorithm. The reference and related parameters of the sets of task graphs are shown in Tables 3 and 5. The schedule lengths of HCPPEFT, STDH, PEFT, and HEFT algorithmss are presented in Figure 9. The results show that the performance of the HCPPEFT algorithm outperforms the other algorithms.

## 5. Conclusion

In this paper, we have proposed a synthesized task scheduling algorithm for heterogeneous computing systems called HCPPEFT. This new algorithm is a two-phase algorithm that combines mechanisms of list-scheduling-based, duplication-based, and look-ahead-based algorithms. Therefore, the HCPPEFT algorithm provides a more efficient way to schedule general task graphs. In the task prioritizing phase, three levels of priority are proposed to choose task. The method of constructing task scheduling queue not only takes account of critical tasks but also takes account of the importance of parent tasks. In the resource selection phase, the duplication of parent tasks is to reduce communication costs. Forecasting the impact of an assignment for all children of the current task is to select a best resource. The effective performance of the new algorithm is compared to three of the best existing scheduling algorithms: HEFT, PEFT, and STDH algorithms. The comparative study is based on randomly generated task graph and the sets of task graphs. The HCPPEFT algorithm outperforms the other algorithms in terms of average SLR, speedup, and efficiency.

## Figures and Tables

**Algorithm 1:**
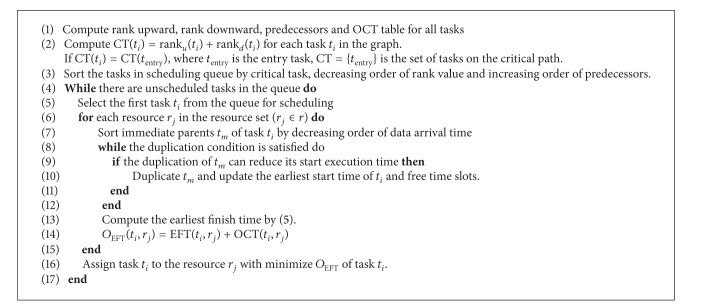
The HCPPEFT algorithm.

**Table 1:** Computation costs.

Task	*r*_1_	*r*_2_	*r*_3_
1	11	19	6
2	18	5	12
3	7	20	13
4	12	16	4
5	19	5	16
6	8	23	14
7	14	24	19
8	7	9	20
9	12	14	16
10	17	7	13

**Table 2:** Values of attributed used in HCPPEFT algorithm for task graph in Figure 2.

*t*_*i*_	rank_*u*_(*t*_*i*_)	rank_*d*_(*t*_*i*_)	rank_*u*_(*t*_*i*_) + rank_*d*_(*t*_*i*_)	Number of predecessors
*t*_1_	105.667	0.000	105.667	0
*t*_2_	73.000	23.000	96.000	1
*t*_3_	69.667	36.000	105.667	1
*t*_4_	57.000	25.000	82.000	1
*t*_5_	76.667	21.000	97.667	1
*t*_6_	58.333	26.000	84.333	1
*t*_7_	50.333	55.333	105.667	3
*t*_8_	36.333	45.667	82.000	2
*t*_9_	36.333	66.333	102.667	3
*t*_10_	12.333	93.333	105.667	3

**Table 3:** Optimistic cost table for the DAG of Figure 2.

Task	*r*_1_	*r*_2_	*r*_3_
*t*_1_	47	51	45
*t*_2_	31	31	32
*t*_3_	31	31	32
*t*_4_	29	29	29
*t*_5_	31	31	32
*t*_6_	28	21	28
*t*_7_	17	7	13
*t*_8_	17	7	13
*t*_9_	17	7	13
*t*_10_	0	0	0

**Table 4:** Schedule produced by the HCPPEFT algorithm in each iteration.

Step	Ready queue	Task selected	EFT			*O*_EFT_			Resource selected
			*r*_1_	*r*_2_	*r*_3_	*r*_1_	*r*_2_	*r*_3_	
1	*t*_1_	*t*_1_	11	19	**6**	58	70	**51**	*r*_3_
2	*t*_3_	*t*_3_	**18**	39	19	**49**	70	51	*r*_1_
3	*t*_5_, *t*_2_, *t*_7_	*t*_5_	37	**20**	22	68	**51**	54	*r*_2_
4	*t*_2_, *t*_7_	*t*_2_	36	25	**18**	67	56	**50**	*r*_3_
5	*t*_7_	*t*_7_	**47**	49	52	64	**56**	65	*r*_2_
6	*t*_4_, *t*_8_, *t*_6_, *t*_9_, *t*_10_	*t*_4_	31	65	**22**	60	94	**51**	*r*_3_
7	*t*_8_, *t*_6_, *t*_9_, *t*_10_	*t*_8_	**37**	58	43	**54**	65	56	*r*_1_
8	*t*_6_, *t*_9_, *t*_10_	*t*_6_	45	72	**36**	73	93	**64**	*r*_3_
9	*t*_9_, *t*_10_	*t*_9_	55	63	**52**	72	70	**65**	*r*_3_
10	*t*_10_	*t*_10_	79	**69**	81	79	**69**	81	*r*_2_

**Table 5:** Reference and related parameters for the sets of task graphs.

Serial number	Number of tasks	Number of resource	CCR	Reference
1	10	3	0.6025	[5, 6, 32]
2	5	3	0.4508	[13]
3	12	4	0.3003	[12]
4	10	3	0.3946	[30]
5	10	3	0.4170	[9]
